# Pure Air

**Published:** 1890-03

**Authors:** 


### PURE AIR.

Pure air of moderate temperature is the prime source of health to the body through action of the blood. It is thus promotive of the change and renewal of structure, of animal heat and of vital energy. It is the grand agent in furthering all the processes of nutrition, it braces the nerves, plants roses on the cheeks, makes the plain look pleasing and the lovely more lovely still.—*Ex.*

